# Induction of Peripheral Tolerance in Ongoing Autoimmune Inflammation Requires Interleukin 27 Signaling in Dendritic Cells

**DOI:** 10.3389/fimmu.2017.01392

**Published:** 2017-10-27

**Authors:** Rodolfo Thomé, Jason N. Moore, Elisabeth R. Mari, Javad Rasouli, Daniel Hwang, Satoshi Yoshimura, Bogoljub Ciric, Guang-Xian Zhang, Abdolmohamad M. Rostami

**Affiliations:** ^1^Department of Neurology, Jefferson Hospital for Neuroscience, Thomas Jefferson University, Philadelphia, PA, United States; ^2^Department of Neurology, Graduate School of Medical Sciences, Neurological Institute, Kyushu University, Fukuoka, Japan

**Keywords:** interleukin 27, tolerance, experimental autoimmune encephalomyelitis, dendritic cells, immunomodulation

## Abstract

Peripheral tolerance to autoantigens is induced *via* suppression of self-reactive lymphocytes, stimulation of tolerogenic dendritic cells (DCs) and regulatory T (Treg) cells. Interleukin (IL)-27 induces tolerogenic DCs and Treg cells; however, it is not known whether IL-27 is important for tolerance induction. We immunized wild-type (WT) and IL-27 receptor (WSX-1) knockout mice with MOG_35–55_ for induction of experimental autoimmune encephalomyelitis and intravenously (i.v.) injected them with MOG_35–55_ after onset of disease to induce i.v. tolerance. i.v. administration of MOG_35–55_ reduced disease severity in WT mice, but was ineffective in *Wsx*^−/−^ mice. IL-27 signaling in DCs was important for tolerance induction, whereas its signaling in T cells was not. Further mechanistic studies showed that IL-27-dependent tolerance relied on cooperation of distinct subsets of spleen DCs with the ability to induce T cell-derived IL-10 and IFN-γ. Overall, our data show that IL-27 is a key cytokine in antigen-induced peripheral tolerance and may provide basis for improvement of antigen-specific tolerance approaches in multiple sclerosis and other autoimmune diseases.

## Introduction

Given that autoimmune diseases result from an imbalance between tolerance and immune response against self-antigens (Ag), induction of Ag-specific immune tolerance is a desired goal in therapy. Peripheral tolerance to innocuous Ags can be achieved by their intravenous (i.v.) administration or *via* mucosal surfaces in non-immunizing conditions. Oral delivery of auto-Ags reduces the severity of autoimmunity in disease models such as collagen-induced arthritis and experimental autoimmune encephalomyelitis (EAE), the prototypical model for human multiple sclerosis (MS) (1–5). i.v. delivery of auto-Ag reduces severity of EAE by stimulating tolerogenic dendritic cells (DCs) and regulatory T (Treg) cells leading to modulation of Ag-presentation (6, 7). Th17, Th1, and memory T cells are suppressed in i.v.-tolerized EAE mice through the modulation of JAK/STAT pathways (8–10). In summary, both oral and i.v. tolerance elicit Ag-specific immunomodulation that relies on stimulation of tolerogenic DCs, Tregs, and augmentation of anti-inflammatory cytokine production (11).

Interleukin (IL)-27 is an anti-inflammatory cytokine that stimulates development of IL-10-producing type 1 regulatory T (Tr1) cells in a STAT-1-dependent pathway (12, 13). Exposure to IL-27 also suppresses Th17 differentiation while stimulating expression of the coinhibitory molecule Tim-3 by T cells (14–16). IFN-γ-primed DCs secrete IL-27 and induce IL-10 production by T cells to reduce EAE (17). WSX-1 (IL-27Rα) and gp130 are subunits of the heterodimeric receptor for IL-27. WSX-1 is expressed in T cells, macrophages, B cells, and DCs (13, 18–20). In DCs, IL-27 signaling induces expression of CD39 and B7-H1, which play a suppressive role in EAE development (21, 22). These observations place IL-27 among key immunomodulatory cytokines; however, the role of IL-27 in peripheral tolerance is not known.

In this study, we investigated the role of IL-27 in peripheral tolerance. *Wsx*^−^*^/^*^−^ mice with EAE were resistant to tolerance induction by i.v. or oral delivery of Ag, and lack of WSX-1 only in T cells did not abrogate tolerance induction. Conversely, transfer of wild-type (WT) DCs to *Wsx*^−^*^/^*^−^ mice rendered them susceptible to i.v. tolerance induction. Transfer of IL-10^−/−^ DCs to *Wsx*^−^*^/^*^−^ mice abrogated i.v. tolerance induction, showing that peripheral tolerance is dependent, at least in part, on IL-27-induced IL-10 production by DCs. Furthermore, by using isolated specific subsets of splenic DCs, our data suggest the existence of a cooperation among spleen DC subpopulations that leads to increased IFN-γ, IL-10, and L-27 production and suppression of IL-17. Altogether, these observations highlight the pivotal role of IL-27 in induction of peripheral tolerance in autoimmune inflammation and provide a basis for future therapeutic approaches aiming to induce tolerance in patients with MS and other autoimmune diseases.

## Materials and Methods

### Mice

Age- and sex-matched WT, RAG-1^−/−^, *Wsx*^−^*^/^*^−^, IL-10^−/−^, and STAT1^−/−^ female mice on the C57BL/6 background were purchased from The Jackson Laboratory (Bar Harbor, ME, USA). Mice were kept in clean cages with a maximum of 5 mice per cage, in a controlled environment with 12/12 h of light/dark cycles and food *ad libitum* throughout the experimental procedures. Every effort was made to minimize suffering of mice. Experimental protocols were approved by the Institutional Animal Care and Use Committee of Thomas Jefferson University.

### EAE Induction and Evaluation

Experimental autoimmune encephalomyelitis was induced as previously described (23). Anesthetized mice were subcutaneously injected with 200 µL of an emulsion containing 200 µg of MOG_35–55_ peptide (MEVGWYRSPFSRVVHLYRNGK, Genscript, NJ, USA) and equal volume of Complete Freund’s adjuvant supplemented with 10 mg/mL of heat-killed *Mycobacterium tuberculosis* H37Ra. Additionally, mice were intraperitoneally injected with 240 ng of pertussis toxin at immunization time and 48 h later. Disease development was analyzed daily and scored on a 0–5 scale: 0—no clinical signs, 1—limp tail, 2—hind limb weakness, 3—hind limb paralysis, 4—hind limb paralysis and front limb weakness, and 5—full paralysis/death. Cumulative scores were calculated as the sum of all daily scores of each individual mouse divided by the number of mice in each group.

### i.v. and Oral Administration of Auto-Ag

Intravenously tolerance was induced as previously described (6). Briefly, each mouse received 200 µg of MOG_35–55_ peptide in 100 µL of PBS at days 14, 17, and 21 postimmunization (p.i.). Control mice received PBS only. Induction of oral tolerance followed a previously described protocol (2), where each mouse was given 200 µg of MOG_35–55_ peptide by oral gavage at days 14, 16, and 18 p.i. and control mice received PBS.

### Ag-Specific Recall Response

Experimental autoimmune encephalomyelitis mice were dissected at day 21 p.i. and draining lymph nodes and spleens were collected in Iscove’s modified Dulbecco’s medium (IMDM), supplemented with 10% heat-inactivated fetal bovine serum, penicillin (100 U), streptomycin (10 µg/mL), l-glutamine (0.3 mg/mL), and 2-mercaptoethanol (55 µM) (referred to as complete IMDM) and disrupted through a 70 µm cell strainer to prepare single cell suspensions. After treatment with RBC lysis buffer (Biolegend, CA, USA) cells were extensively washed with complete IMDM by centrifugation at 1,300 rpm for 5 min at 4°C and the cell density was adjusted to 2 million cells/mL. 100 µL of adjusted cell suspension was added to each well of a 96 well plate. The same volume of MOG_35–55_ (100 µg/mL) was added to wells to a final concentration of 50 µg/mL of antigen. Cells were incubated at 37°C for 72 h. For negative control, cells were cultured without Ag, while cells treated with anti-CD3 and anti-CD28 (1 µg/mL each) served as a positive control. After the incubation period, supernatants were collected and stored at −20°C until use and cells were analyzed for proliferation by flow cytometry.

### Cytokine Detection in Culture Supernatants

Supernatants from cell cultures were kept at −20°C until use. Cytokine concentrations in culture supernatants were measured with sandwich enzyme-linked immunosorbent assay (ELISA) using commercial kits, following the manufacturer’s recommendation (R&D Systems, Minneapolis, MN, USA). The following cytokines were measured: IFN-γ, IL-17, IL-10, IL-27, and GM-CSF.

### Isolation of CNS Cells and Flow Cytometry

Mononuclear cells from the CNS of EAE mice were isolated by Percoll gradient centrifugation. In brief, mice were euthanized and perfused with ice-cold PBS. Brains and spinal cords were removed and placed in IMDM, mechanically dissociated with scissors and enzymatically digested by incubation with 700 µg/mL Liberase TL (Sigma-Aldrich, USA) at 37°C for 30 min. The preparation was then washed in IMDM, and the pellet was fractionated on a 70/30% Percoll gradient. MNCs were recovered from the interface, washed, and resuspended in IMDM/10% FBS. One million cells were transferred to FACS tubes. CNS cells were stimulated with PMA (50 ng/mL) and ionomycin (500 ng/mL) in the presence of GolgiPlug (1 μg/10^6^ cells) for 4 h at 37°C. Cells were washed in FACS buffer (PBS/2% FBS) and stained with fluorochrome-labeled Abs to CD45 (Clone 30-F11), CD4 (Clone GK1.5), CD11c (Clone N418), CD11b (Clone M1/70), CD103 (Clone 2E7), MHC-II (Clone M5/114.15.2), CD86 (Clone PO3), and IL-27Rα (Clone 2918, BD Biosciences) for 20 min at 4°C in a final volume of 100 µL. Cells were washed, fixed, and permeabilized using Fix and Perm cell permeabilization reagents (eBioscience). Cells were intracellularly stained for IL-10 (Clone JES5-16E3), IL-17 (Clone TC11-18H10.1), IL-27 (Clone MM27-7B1), GM-CSF (Clone MP1-22E9, BD Biosciences), IFN-γ (XMG 1.2), or Foxp3 (Clone FJK-16s, eBioscience) for 30 min at 4°C in a final volume of 100 µL. Unless otherwise stated, all antibodies were from Biolegend, CA. Data were acquired on a FACSAria (BD Bioscience) and analyzed using FlowJo Software (Tristar Inc.).

### Reconstitution of *Wsx*^−/−^ Mice and EAE Induction

*Wsx*^−^*^/^*^−^ mice received magnetic bead-isolated CD11c^+^ DCs from spleens of WT, IL-10^−/−^, and *Wsx*^−^*^/^*^−^ mice. Each mouse received 1 million CD11c^+^ DCs by i.v. injection under anesthesia. After 72 h of adoptive transfer, anesthetized reconstituted *Wsx*^−^*^/^*^−^ mice were immunized with MOG_35–55_ peptide in CFA to induce EAE, as described above. Disease development was evaluated daily.

### Reconstitution of RAG1^−/−^ Mice and EAE Induction

RAG1^−/−^ mice were reconstituted with magnetic bead-isolated total CD4^+^ T cells from spleens of WT and *Wsx*^−^*^/^*^−^ mice. Each mouse received 2 million CD4^+^ T cells *via* i.v. injection under anesthesia. After 72 h of adoptive transfer, EAE was induced in reconstituted mice as described above.

### OVA Immunization and Treatment

Anesthetized female WT C57BL/6 mice were subcutaneously injected with 100 µL of an emulsion containing 100 µg of OVA (grade V, Sigma-Aldrich, St. Louis, MO, USA) and an equal volume of CFA supplemented with 4 mg/mL of Heat-Killed *Mycobacterium tuberculosis* H37Ra. Seven days later, mice were i.v. given 100 µL of OVA-Alexa Fluor 647 (Thermo Fisher Scientific, MA, USA). Mice were dissected after 2 h of OVA injection, and spleens, draining, and mesenteric lymph nodes were collected, stained, and analyzed by flow cytometry.

### DC Sorting and Culture

Splenic CD11c^+^ DCs were isolated from spleens from MOG_35–55_-tolerized and non-tolerized EAE mice using magnetic beads (CD11c^+^ cell isolation kit, Miltenyi Biotec, CA, USA). Isolated DCs were surface stained with antibodies against CD11c, CD11b, and CD103 for 20 min at 4°C and then sorted into four CD11c^+^ populations: CD11b^−^CD103^−^, CD11b^+^CD103^−^, CD11b^+^CD103^+^, and CD11b^hi^CD103^+^. Sorting was performed on FACSAria III (BD Biosciences, CA, USA). Sorted DCs were plated in U-bottom 96 well plates at a density of 20,000 cells/well. For Ag-presentation assays, naive CD4^+^ T cells from WT mice were isolated using magnetic beads (Naive CD4^+^ T cell isolation kit, Miltenyi Biotec, CA, USA). Isolated T cells were CD3^+^CD4^+^CD44^−^CD62L^+^ as analyzed by flow cytometry. 200,000 naive CD4^+^ T cells were added to each well of the cell culture plate containing DCs (ratio of 1 DC: 10 T cells) and plates were incubated at 37°C in the presence of anti-CD3 (0.5 µg/mL). Cells were collected after 72 h and analyzed by flow cytometry, while cytokine concentrations in culture supernatants were measured by ELISA.

### Generation of Bone Marrow-Derived DCs

Dendritic cells were generated from bone marrow precursors, according to a previously described protocol (24, 25). Briefly, precursors from WT mice were seeded at 1 million cells/mL in Petri dish in complete IMDM supplemented with 10 ng/mL of GM-CSF. Culture medium was changed on days 3 and 6. At day 7, cells were collected, washed in fresh complete media and transferred to appropriate culture plates for specific treatments. Maturation of the DCs was induced with lipopolysaccharide (100 ng/mL) and cells were cultured in the presence/absence of IL-27 (20 ng/mL) for 18 h and used in Ag-presentation assays or adoptive transfer experiments.

### Statistical Analysis

Daily clinical scores among experimental groups in EAE were compared by two-way ANOVA and posttested with Sidak. Analyses between three or more groups were performed with one-way ANOVA and posttested with Bonferroni. Comparisons between two groups were carried out with Student’s *t*-test. Values of *p* < 0.05 were considered significant.

## Results

### IL-27 Is Necessary for Tolerance Induction in EAE

**Figure 1:**
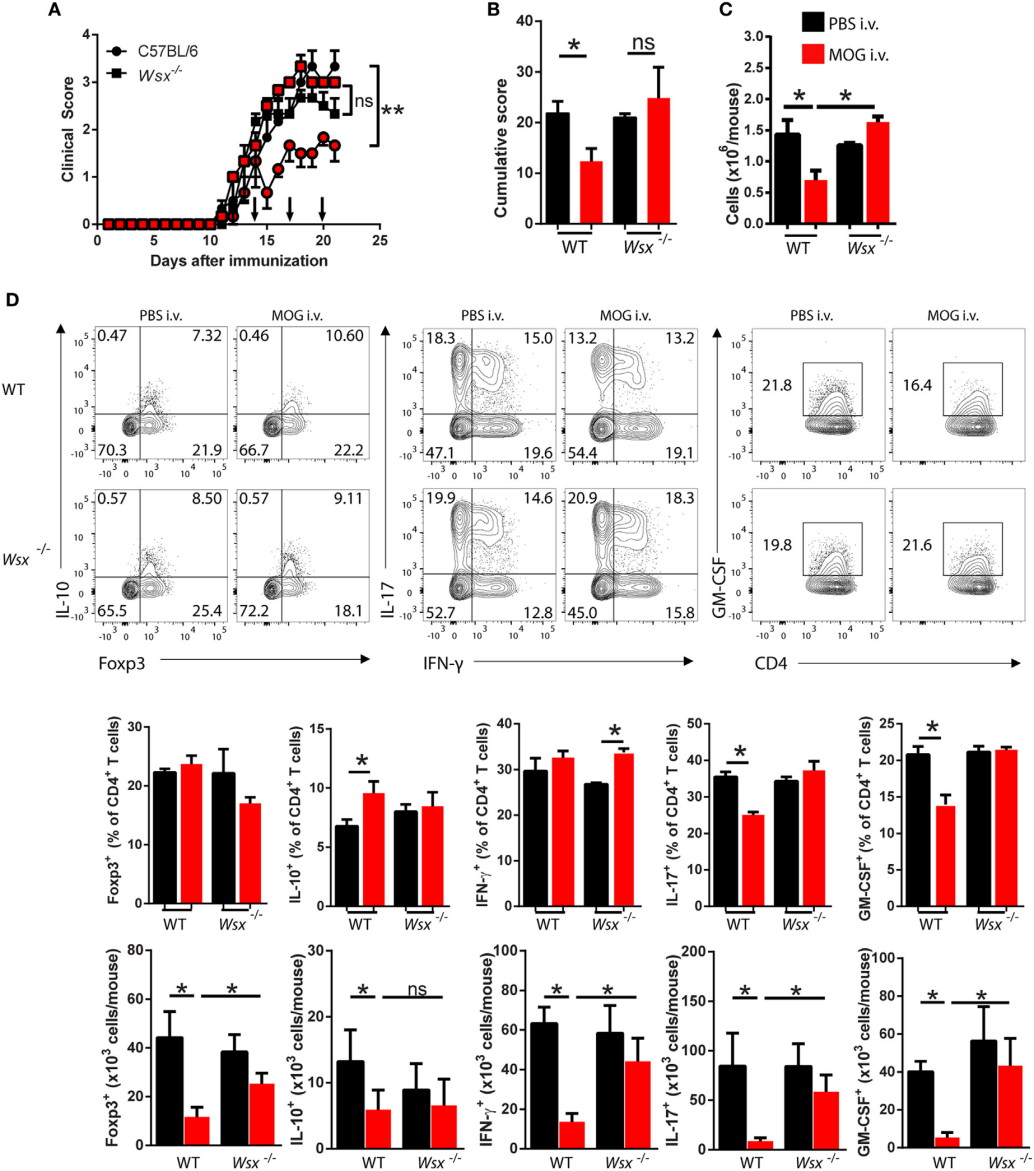

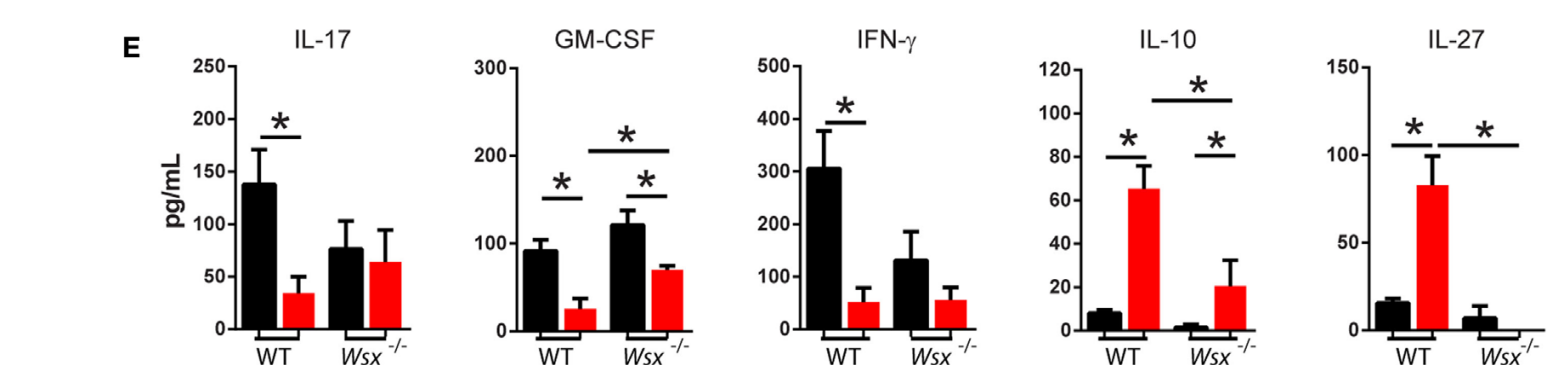
Lack of interleukin (IL)-27Rα precludes the therapeutic effect of intravenous (i.v.) MOG_35–55_. Wild-type (WT) and *Wsx*^−^*^/^*^−^ mice (*n* = 5 per group) were immunized with MOG_35–55_ to induce experimental autoimmune encephalomyelitis (EAE). At days 14, 17, and 20 postimmunization (p.i.), mice were i.v. injected with 200 µg of MOG_35–55_ to induce tolerance. **(A)** Daily clinical scores of disease severity. **(B)** Cumulative scores of disease severity. **(C)** Mice were dissected at day 21 p.i. and CD45^+^ CNS-infiltrating cells numbers were determined by flow cytometry and hemocytometer. **(D)** Flow cytometry analysis of IL-10^+^, Foxp3^+^, IL-17^+^, GM-CSF^+^, and IFN-γ^+^ CD4^+^ T cells from the CNS of EAE mice described above. **(E)** Cytokine concentrations in supernatants from cultures of spleen cells from EAE mice were determined by ELISA. Results are representative of three independent experiments with similar outcomes. Bar graphs depict mean ± standard error mean (SEM). Values of *p* < 0.05 (*), *p* < 0.01 (**) were considered statistically significant. Two-way ANOVA [in **(A)**] and one-way ANOVA **(B,C,E)** with Bonferroni posttest were used to analyze data.

We first tested whether IL-27 is necessary for induction of tolerance in EAE. *Wsx*^−^*^/^*^−^ and WT C57BL/6 mice were immunized with MOG_35–55_ peptide to induce EAE. At day 14 p.i., when clinical disease had already developed, mice received i.v. 200 µg of MOG_35–55_ and treatment continued with two additional doses of MOG_35–55_ 3 days apart. While, compared with PBS-treated controls, MOG_35–55_ treatment significantly reduced clinical score in WT mice, it had little effect in *Wsx*^−^*^/^*^−^ mice (Figures 1A,B). MOG_35–55_-treated mice had fewer leukocytes in the CNS compared to PBS-treated WT mice and MOG_35–55_- and PBS-treated *Wsx*^−^*^/^*^−^ mice (Figure 1C). i.v. injection of MOG_35–55_ increased the frequencies of IL-10^+^ CD4^+^ T cells while inhibiting IL-17- and GM-CSF-producing T cells in the CNS of WT, but not of *Wsx*^−^*^/^*^−^ mice (Figure 1D). No significant differences were observed in total frequency of Foxp3^+^CD4^+^ T cells (Figure 1D). However, due to lower numbers of infiltrating cells in the CNS of WT mice treated with MOG_35–55_, absolute numbers of IL-10^+^, Foxp3^+^, IFN-γ^+^, IL-17^+^, and GM-CSF^+^ CD4^+^ T cells were reduced in these mice compared to other groups (Figure 1D). In addition, cytokine production from Ag-specific *ex vivo* re-stimulated spleen cells from WT MOG-treated mice revealed a shift toward IL-10 and IL-27 production and suppression of IL-17, GM-CSF and IFN-γ production compared to cells from PBS-treated mice, whereas such a pattern was not observed in cultures from *Wsx*^−^*^/^*^−^ mice (Figure 1E). In summary, i.v. tolerance in EAE is followed by a modulation of the immune response in the CNS and secondary lymphoid organs in an IL-27-dependent manner.

In order to investigate whether IL-27 is also necessary for oral tolerance induction, WT and *Wsx*^−^*^/^*^−^ EAE mice were fed with three 200 µg doses of MOG_35–55_ every other day starting at disease onset. Similar to the i.v. tolerance model, WT mice fed with MOG_35–55_ developed significantly less severe EAE compared to PBS-fed mice, but this effect was not observed in *Wsx*^−^*^/^*^−^ mice (Figure S1A in Supplementary Material). Significantly fewer cells infiltrated the CNS of MOG_35–55_-fed WT mice compared with controls, whereas there was no difference between test and control groups of *Wsx*^−^*^/^*^−^ mice (Figure S1A in Supplementary Material). MOG-fed WT mice also had similar frequencies of IL-10-producing Foxp3^+^ Treg cells and IFN-γ-, IL-17-, and GM-CSF-producing CD4^+^ T cells compared to *Wsx*^−^*^/^*^−^ mice and PBS-fed controls (Figure S1B in Supplementary Material). DC maturation was suppressed in MOG-fed WT mice, but not in other groups (Figure S1C in Supplementary Material). As previously described by our group (6), DCs from i.v. tolerized mice also show reduced expression of Ag-presenting molecules (data not shown). Interestingly, oral tolerance seems less effective than i.v. tolerance in suppressing EAE. We speculate that MOG peptide is readily available to APCs and T cells following i.v. administration, whereas gastric acid and oral mucosa barrier may interfere with the bioavailability of orally delivered peptides. We believe that oral and i.v. administration of peptides leads to tolerance through different pathways that converge in IL-27R signaling as both approaches (i.v. and oral tolerance) failed to suppress disease in *Wsx*^−^*^/^*^−^ mice. Taken together, these observations indicate that IL-27 is a key cytokine for inducing peripheral tolerance *via* i.v. and oral route.

### IL-27 Signaling in T Cells Is Dispensable for Induction of Peripheral Tolerance

**Figure 2:**
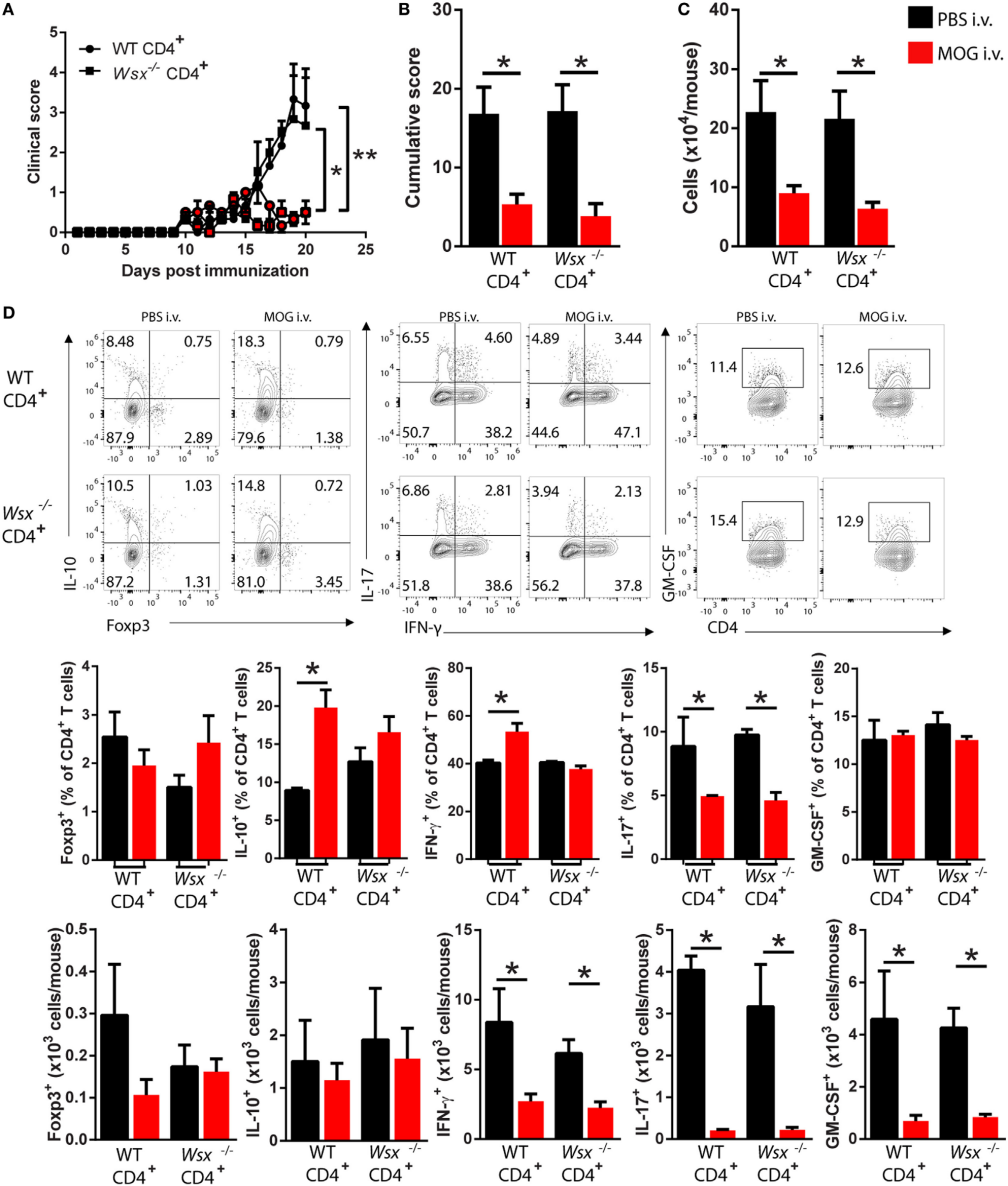
Absence of interleukin (IL)-27Rα in T cells does not preclude i.v. tolerance induction. RAG1^−/−^ mice were reconstituted with total CD4^+^ T cells from wild-type (WT) or *Wsx*^−^*^/^*^−^ mice. 72 h postreconstitution, recipient mice were immunized for EAE induction and MOG_35–55_ (200 μg/dose) was given i.v. at days 14, 17, and 20 postimmunization (p.i.). **(A)** Daily clinical scores of disease severity. **(B)** Cumulative scores of disease severity. **(C)** Mice were dissected on day 21 p.i. and CD45^+^ CNS-infiltrating cells numbers were determined by flow cytometry and hemocytometer. **(D)** Flow cytometry analysis of IL-10^+^, Foxp3^+^, IL-17^+^, GM-CSF^+^, and IFN-γ^+^ CD4^+^ T cells from the CNS of EAE mice described above. Bar graphs depict mean ± SEM. Values of *p* < 0.05 (*), *p* < 0.01 (**) were considered statistically significant. Two-way ANOVA [in **(A)**] and one-way ANOVA [in **(B)**] with Bonferroni posttest were used for data analyses. Data are representative of two independent experiments with *n* ≥ 3 mice per group with similar outcomes.

To determine whether IL-27 signaling in T cells plays a role in tolerance induction, we reconstituted RAG1^−/−^ mice with WT or *Wsx*^−^*^/^*^−^ CD4^+^ T cells and immunized them for EAE induction. Mice were i.v. injected with 200 µg of MOG_35–55_ on days 14, 17, and 20 p.i. Both RAG1^−/−^ mice reconstituted with WT and with *Wsx*^−^*^/^*^−^ CD4^+^ T cells and i.v. tolerized developed significantly milder disease than their PBS-treated counterparts (Figures 2A,B). i.v. administration of MOG_35–55_ significantly reduced numbers of infiltrating cells in the CNS of mice that were reconstituted with WT and *Wsx*^−^*^/^*^−^ CD4^+^ T cells (Figure 2C). Among infiltrating cells, we observed a reduction in frequency of IL-17^+^ T cells irrespective of WSX expression in T cells (Figure 2D). Frequencies of IL-10^+^ and IFN-γ^+^ T cells were increased only in i.v. tolerized RAG1^−/−^ mice reconstituted with WT CD4^+^ T cells (Figure 2C). However, due to lower numbers of infiltrating cells in the CNS of mouse treated with MOG_35–55_, absolute numbers of IFN-γ^+^, IL-17^+^, and GM-CSF^+^ CD4^+^ T cells were reduced in these mice compared to PBS-treated groups (Figure 1D). Interestingly, IL-10^+^ and Foxp3^+^ CD4^+^ T cell numbers were comparable between PBS and MOG-treated groups (Figure 2D). Tolerized mice also had a higher frequency of spleen Foxp3^+^ Treg cells compared to PBS-treated mice, irrespective of WSX expression (data not shown). These results suggest that IL-27 signaling in T cells is not necessary for immune modulation after i.v. delivery of auto-Ag.

### IL-27Rα Expression by DCs Is Required for Peripheral Tolerance Induction *via* a Mechanism That Is Dependent on IL-10 Production by DCs

**Figure 3:**
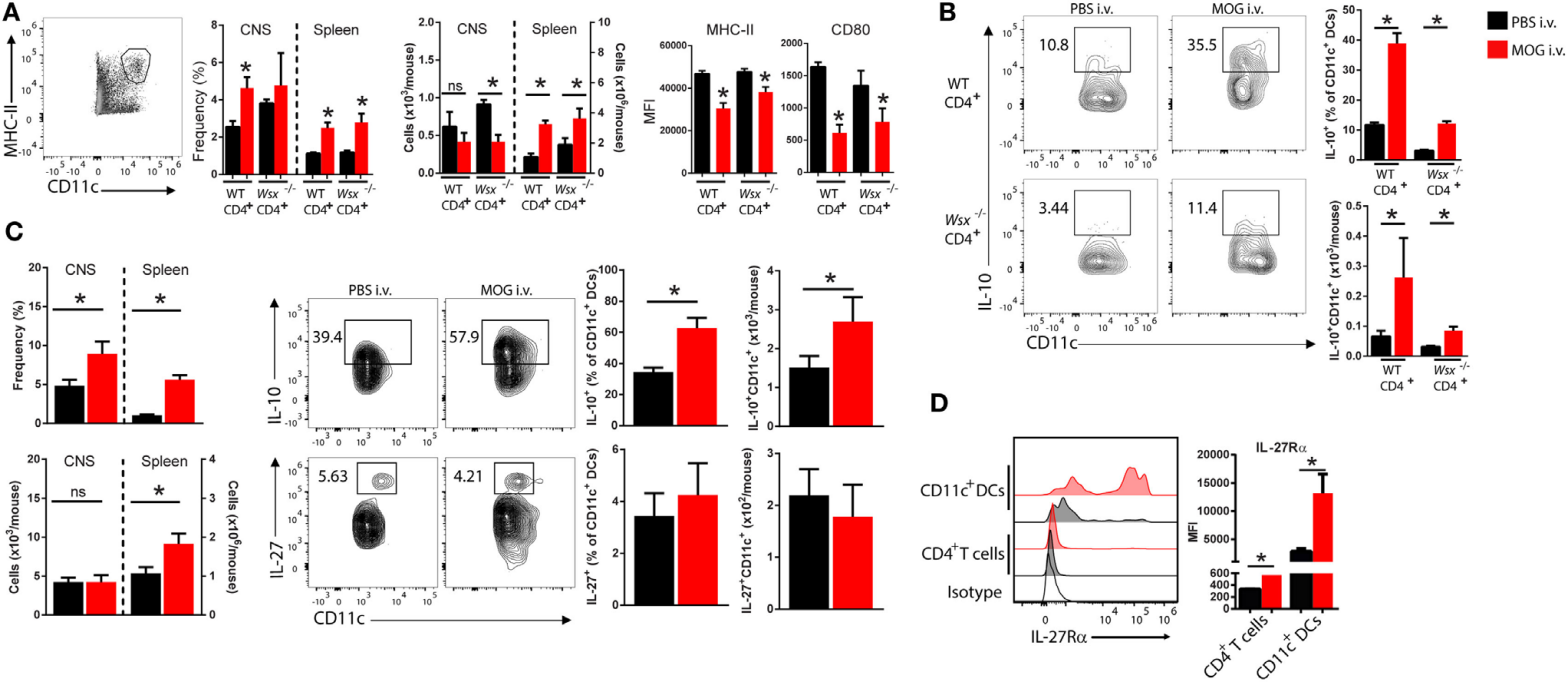
Changes in dendritic cells (DCs) frequency and phenotype in i.v. tolerized mice. **(A)** RAG1^−/−^ reconstituted with total CD4^+^ T cells from wild-type (WT) or *Wsx*^−^*^/^*^−^ mice were immunized to induce experimental autoimmune encephalomyelitis (EAE). At day 21, postimmunization (p.i.) mice were dissected and analyzed for frequency and phenotype of DCs. Flow cytometry analysis of frequency of DCs (CD11c^+^MHC II^+^) and expression of MHC class II and CD80 expression on DCs from the CNS and spleens of mice. **(B)** IL-10 production by DCs from the CNS of EAE mice. **(C)** Frequency and absolute numbers of DCs in CNS and spleens of WT EAE mice and detection of IL-10 and IL-27 production by DCs. **(D)** IL-27Rα expression in T cells and DCs from the CNS of WT EAE mice at day 21 p.i. Representative data from three independent experiments. Bar graphs depict mean ± SEM. Values of *p* < 0.05 (*) were considered statistically significant. NS, not significant.

Given that DCs also express WSX, we then analyzed the frequency and phenotype of DCs in the CNS of RAG-1^−/−^ mice reconstituted with WT and *Wsx*^−^*^/^*^−^ CD4^+^ T cells. i.v. tolerized mice had a higher frequency of DCs in the CNS and these cells expressed less MHC class II and CD80 (Figure 3A). DCs from i.v. tolerized mice also produced higher levels of IL-10 compared to PBS-treated mice (Figure 3B). Of note, DCs from i.v. tolerized mice that received *Wsx*^−^*^/^*^−^ T cells had fewer IL-10^+^ DCs compared to i.v. tolerized mice that were reconstituted with WT T cells (Figure 3B). These observations indicate that IL-27 signaling in non-CD4^+^ T cells, such as DCs, is required for induction of Ag-specific tolerance in ongoing inflammation.

We then investigated the role of IL-27 signaling in DCs in i.v. tolerance induction. CNS-infiltrating DCs obtained from i.v. MOG_35–55_-treated WT mice with EAE had higher IL-10 production compared to PBS-treated controls (Figure 3C). No significant change in IL-27 production was observed between DCs from PBS and MOG_35–55_-treated groups (Figure 3C). DCs from CNS of i.v. tolerized mice also showed lower expression levels of MHC-II, CD80, and CD86 molecules compared to PBS-treated counterparts (data not shown). Interestingly, DCs in the CNS expressed significantly higher levels of IL-27Rα compared to T cells, and its expression was increased in i.v. tolerized mice (Figure 3D). These observations suggested that DCs may be relevant to the role of IL-27 in tolerance induction.

**Figure 4:**
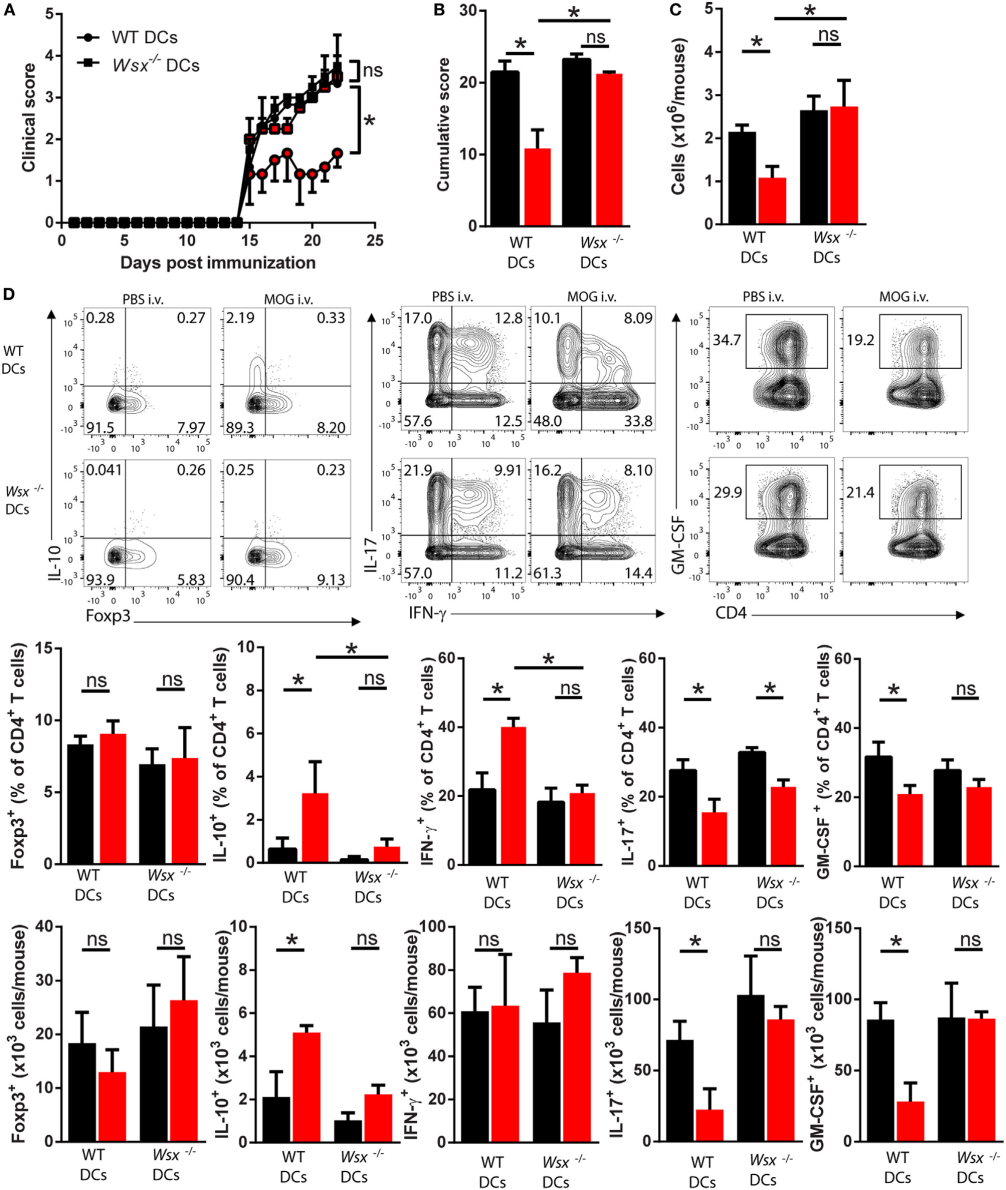
Interleukin (IL)-27 signaling in dendritic cells (DCs) is required for intravenous (i.v.) tolerance induction in ongoing experimental autoimmune encephalomyelitis (EAE). *Wsx*^−^*^/^*^−^ mice were reconstituted with wild-type (WT) and *Wsx*^−^*^/^*^−^ CD11c^+^ DCs and 72 h later were immunized to induce EAE. EAE mice received i.v. MOG at days 14, 17, and 20 postimmunization (p.i.) to induce tolerance. Clinical **(A)** and cumulative **(B)** scores are shown. **(C)** Mice were dissected on day 21 p.i. and CD45^+^ CNS-infiltrating cells numbers were determined by flow cytometry and hemocytometer. **(D)** Flow cytometry analysis of IL-10^+^, Foxp3^+^, IL-17^+^, GM-CSF^+^, and IFN-γ^+^ CD4^+^ T cells from the CNS of EAE mice described above. Bar graphs depict mean ± SEM. Data are from three independent experiments with *n* of 5 mice per group with similar outcomes. *p < 0.05 [Student’s *t*-test in **(A)**; two-way ANOVA in **(B)**, one-way ANOVA in **(C)**]. ns, not significant.

To test this possibility, we reconstituted *Wsx*^−^*^/^*^−^ mice with splenic CD11c^+^ DCs from WT mice, and as controls we transferred *Wsx*^−^*^/^*^−^ DCs to *Wsx*^−^*^/^*^−^ mice. In this system, a fraction among DCs in *Wsx*^−^*^/^*^−^ mice could respond to IL-27 as they express IL-27Rα. Reconstituted mice were then immunized for EAE induction and subsequently subjected to i.v. tolerance induction. *Wsx*^−^*^/^*^−^ mice reconstituted with WT DCs developed significantly less severe EAE after i.v. MOG_35–55_ treatment, whereas disease in *Wsx*^−^*^/^*^−^ mice that received *Wsx*^−^*^/^*^−^ DCs was not influenced by i.v. tolerization (Figures 4A,B). Analysis of CNS-infiltrating T cells from mice that received WT DCs showed that disease amelioration correlated with a reduced infiltration of cells in the CNS (Figure 4C). Mice recipient of WT DCs showed an increase in the frequencies of IFN-γ- and IL-10-producing T cells and a reduction in frequency of IL-17- and GM-CSF-producing T cells (Figure 4D). On the other hand, mice that had received *Wsx*^−^*^/^*^−^ DCs had only minor changes in the frequencies of cytokine-producing T cells in the CNS, although a significant reduction of IL-17^+^ T cells was observed (Figure 4D). Due to a lower infiltration of cells in the CNS of i.v. tolerized mice recipient of WT DCs, absolute numbers of IL-10^+^ and IFN-γ^+^ CD4^+^ T cells did not show significant differences compared to their PBS-treated counterparts (Figure 4D). Still, absolute numbers of IL-17^+^ and GM-CSF^+^ CD4^+^ T cells were reduced in i.v. tolerized mice recipient of WT DCs compared to PBS-treated counterparts (Figure 4D). In summary, these data show that tolerance induction in ongoing inflammation requires IL-27 signaling in DCs, leading to disease suppression by augmenting IFN-γ and IL-10 production and dampening Th17 and GM-CSF^+^ CD4^+^ T cells.

Given that STAT1 is the principal signaling molecule downstream of IL-27R in T cells, we tested whether STAT1 is also involved in IL-27-mediated DC modulation. Immature and mature bone marrow-derived DCs were treated with IL-27 for 18 h and their expression of surface markers involved with Ag-presentation was evaluated. Consistent with data from the literature (21), IL-27-treated mature WT DCs had reduced expression of MHC class II, CD80, and CD86 (Figure S2A in Supplementary Material). When cultured with naive T cells, IL-27-treated WT DCs suppressed GM-CSF production while inducing IL-10 by T cells (Figures S2B,C in Supplementary Material, respectively). IL-27-treated STAT1^−^ (S1^−^) DCs had a similar pattern of surface molecule expression as WT DCs (Figure S2A in Supplementary Material) and also suppressed GM-CSF production and induced IL-10 in T cells (Figures S2B,C in Supplementary Material, respectively). WT and S1^−^ IL-27-treated DCs stimulated IFN-γ production by T cells (Figures S2B,C in Supplementary Material), which is consistent with our findings of more IFN-γ-producing T cells in the CNS of i.v. tolerized WT DC-reconstituted *Wsx*^−^*^/^*^−^ mice (Figure 4D). We did not detect IL-17 in this culture system (data not shown). Upon adoptive transfer into EAE mice, IL-27-treated WT and S1^−^ DCs ameliorated clinical disease and reduced cellular infiltration in the CNS (Figure S2D in Supplementary Material). Analysis of CD45^+^CD3^+^CD4^+^ T cells that infiltrated the CNS of mice transferred with IL-27-treated WT and S1- DCs showed higher frequency of Foxp3^+^ and IL-10^+^ CD4^+^ T cells (Figure S2E in Supplementary Material). We observed a reduction in frequency of IFN-γ^+^, IL-17^+^, and GM-CSF^+^ CD4^+^ T cells only in recipients of IL-27-treated WT DCs, but not in recipients of S1^−^ DCs (Figure S2E in Supplementary Material). Absolute numbers of CNS infiltrating IFN-γ^+^, IL-17^+^, and GM-CSF^+^ CD4^+^ T cells were reduced in mice recipient of IL-27-treated WT and S1^−^ DCs (Figure S2E in Supplementary Material). These data show that STAT1 signaling is not necessary for IL-27-induced DC modulation.

**Figure 5:**
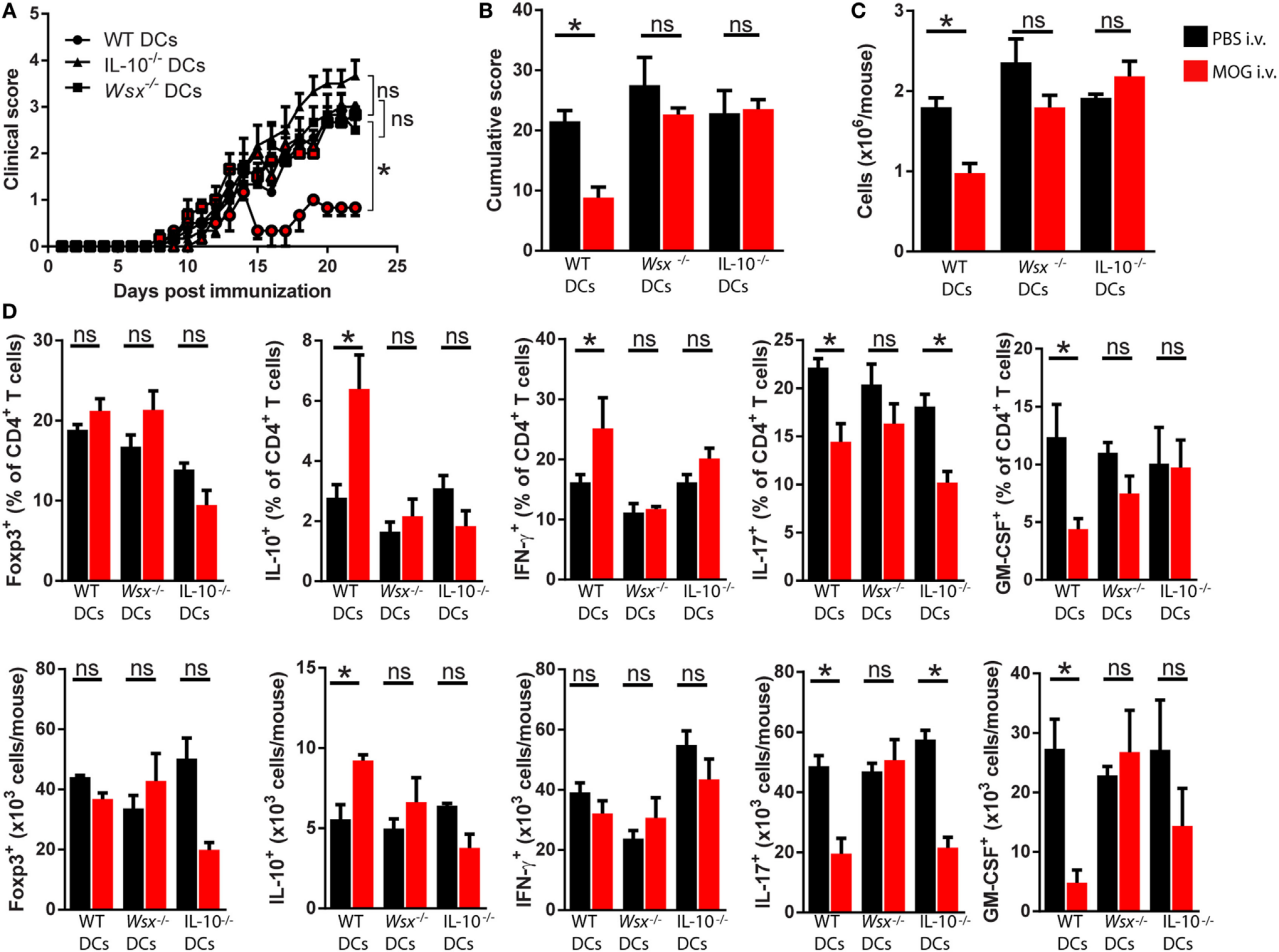
Interleukin (IL)-27-induced dendritic cell (DC)-derived IL-10 is necessary for peripheral tolerance induction. *Wsx*^−^*^/^*^−^ mice were reconstituted with wild-type (WT), *Wsx*^−^*^/^*^−^ or IL-10^−/−^ CD11c^+^, DCs and 72 h later immunized to induce experimental autoimmune encephalomyelitis (EAE). EAE mice received intravenous (i.v.) MOG at days 14, 17, and 20 p.i. to induce tolerance. Clinical **(A)** and cumulative **(B)** scores are shown. **(C)** Mice were dissected on day 21 p.i. and CD45^+^ CNS-infiltrating cells numbers were determined by flow cytometry and hemocytometer. **(D)** Frequency and absolute numbers of CNS-infiltrating GM-CSF^+^, IL-17^+^, IFN-γ^+^, IL-10^+^, and Foxp3^+^ CD4^+^ T cells. Bar graphs depict mean ± SEM. Data are from three independent experiments with *n* of 5 mice per group with similar outcomes. *p < 0.05 [Student’s *t*-test in **(A)**; two-way ANOVA in **(B)**, one-way ANOVA in **(C)**]. ns, not significant.

Next, we tested whether IL-10 production by DCs plays a role in induction of peripheral tolerance. To that end, we reconstituted *Wsx*^−^*^/^*^−^ mice with WT, *Wsx*^−^*^/^*^−^, or IL-10^−/−^ CD11c^+^ DCs and subjected EAE mice to i.v. tolerance induction. Lack of IL-10 production by DCs reduced the protective effect of i.v. MOG_35–55_ administration (Figures 5A,B). Analysis of CNS-infiltrating cells showed that only mice recipient of WT DCs had a reduction in total cell numbers (Figure 5C). Analysis of CD45^+^CD3^+^CD4^+^ T cells that infiltrated the CNS of mice recipient of IL-10^−/−^ DCs showed a reduction infrequency and numbers of IL-17^+^ T cells compared to PBS-treated counterparts (Figure 5D). No significant differences were observed in frequency and numbers of Foxp3^+^, IL-10^+^, IFN-γ^+^, and GM-CSF^+^ CD4^+^ T cells in the CNS of mice recipient of IL-10^−/−^ DCs compared to their PBS-treated counterparts (Figure 5D). Taken together, these observations show that induction of peripheral tolerance in ongoing inflammation relies on IL-27 signaling, which through IL-10-dependent and STAT1-independent mechanisms, modifies DCs to promote IFN-γ and IL-10 production by T cells in the CNS of EAE mice.

### Specific Subsets of Splenic DCs from i.v. Tolerized Mice Stimulate IFN-γ, IL-10, and IL-27 Production in an IL-27-Dependent Manner

**Figure 6:**
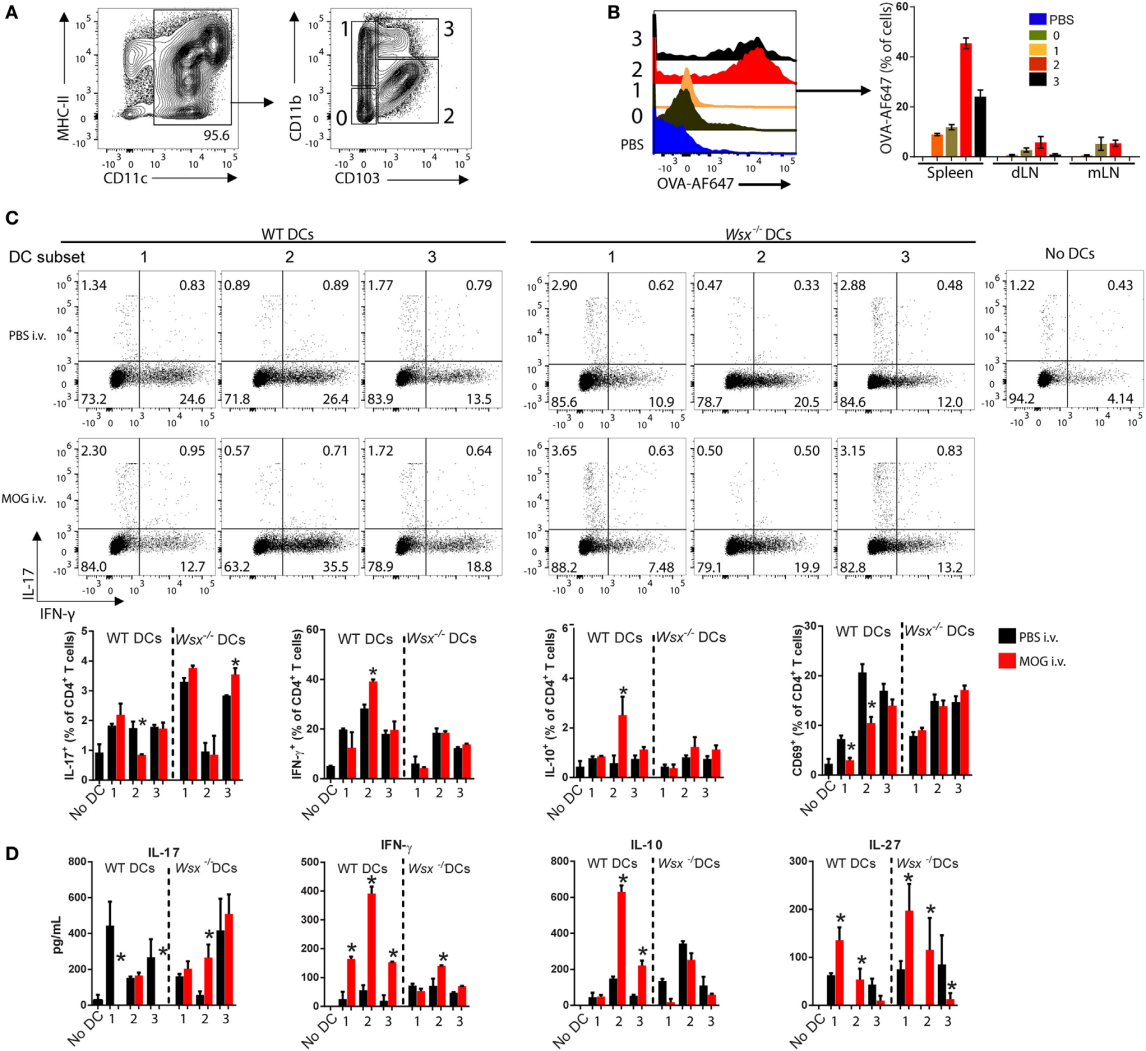
Specific subsets of splenic dendritic cells (DCs) from intravenously (i.v.) tolerized mice induce IFN-γ, interleukin (IL)-10, and IL-27 production in an IL-27-dependent manner. **(A)** Flow cytometry analysis of CD11c^+^ DCs subpopulations isolated from spleens of naive mice. **(B)** OVA-AF647 uptake by DC subsets in spleen, mesenteric lymph node, and draining lymph node 2-h post i.v. injection. **(C)** FACS-sorted DCs were cultivated with wild-type (WT) T cells and anti-CD3 (0.5 µg/mL) at 37°C. After 72 h, cells were analyzed by flow cytometry for expression of cytokines. **(D)** Supernatants were assayed by ELISA. Bar graphs depict mean ± SEM. Data are representative from three independent experiments with *n* ≥ 5 mice per group with similar outcomes. *p < 0.05 (Student’s *t*-test). Bars depict Mean ± SEM.

Given that DCs comprise multiple cell types with distinct functions in immunity, infection and tolerance (26–31), we tested whether a particular DC type has a prominent role in capturing auto-Ag and stimulating T cell response. Based on CD11b and CD103 expression in isolated CD11c^+^ DCs, we distinguished four DC populations (CD11b^−^CD103^−^, CD11b^+^CD103^−^, CD11b^+^CD103^+^, and CD11b^hi^CD103^+^) from the spleen of WT mice (Figure 6A). To determine whether a particular DC subpopulation captures Ag in the course of inflammation, we subcutaneously immunized WT mice with OVA/CFA and i.v. injected Alexa Fluor 647-conjugated OVA (OVA-AF647) 7 days later. Two hours after OVA-AF647 injection, spleens, draining, and mesenteric lymph nodes were collected and assessed for AF647 fluorescence in each DC subset by flow cytometry. OVA-AF647 was primarily captured by CD11b^hi^CD103^+^ and CD11b^+^CD103^+^ DCs in spleens and, to a lesser extent, in dLN and mLN (Figure 6B), suggesting that splenic CD103^+^ DCs predominantly capture i.v. delivered Ags.

We then investigated whether CD103^+^ DCs from tolerized EAE mice are responsible for T cell modulation. WT and *Wsx*^−^*^/^*^−^ EAE mice were i.v. tolerized and DC subsets from spleens sorted and cultured with naive CD4^+^ T cells (1 DC: 10 T ratio) for 72 h. CD11b^−^CD103^−^ cells did not express CD80, CD86 and MHC-II and were unable to stimulate T cells *in vitro* (data not shown); therefore, this subpopulation was excluded from further analysis. CD11b^+^CD103^+^ DCs from i.v. tolerized WT mice were potent inducers of IFN-γ and IL-10 production compared to other DC subpopulations (Figures 6C,D). CD11b^+^CD103^−^ and CD11b^hi^CD103^+^ DCs from WT i.v. tolerized mice suppressed IL-17 production by T cells completely measured by ELISA, even though flow cytometry analysis did not show such suppression pattern (Figures 6C,D). Corresponding *Wsx*^−^*^/^*^−^ DCs increased IL-17 production (Figures 6C,D). Expression of the activation marker CD69 in cultured T cells was significantly lower when cells were cultured with CD11b^+^CD103^−^ and CD11b^+^CD103^+^ DCs from i.v. tolerized WT mice compared to their PBS-treated counterparts and *Wsx*^−^*^/^*^−^ DCs (Figure 6C). IL-27 production was upregulated in co-cultures with CD11b^+^CD103^−^ DCs from MOG-treated WT mice compared to other DC subtypes and DCs from *Wsx*^−^*^/^*^−^ mice (Figure 6D). Interestingly, *Wsx*^−^*^/^*^−^ DCs failed to stimulate production of IFN-γ and IL-10 but efficiently produced IL-27 (Figure 6D). Taken together, our data show that IL-27 induces IL-10 production by DCs to establish peripheral tolerance in ongoing inflammation and that distinct subpopulations of splenic DCs cooperate in this phenomenon.

## Discussion

Ag-specific tolerance is a common goal in transplantation and autoimmune therapies. Peripheral tolerance induced against self and foreign Ags may be achieved by delivering them through “tolerogenic routes” such as nasal, oral, and i.v. (1, 6). Peripheral tolerance is associated with a reduced inflammatory profile of Th cells and a high activity of Tregs and tolerogenic DCs (2, 3, 6, 9). Cytokines, growth factors and glycoproteins are also involved in peripheral tolerance (32, 33). It has been shown that IL-27 subunits and its receptor are upregulated in EAE (34). Given that IL-27 is an anti-inflammatory cytokine that induces IL-10 production by T cells and suppresses EAE (12, 35, 36), we hypothesized that IL-27 plays a role in induction of peripheral tolerance. In this study we show that IL-27 is necessary to establish peripheral tolerance in ongoing autoimmune inflammation. Our data also show that tolerance induction is characterized by reduced infiltration of cells in the CNS, with greater proportions of IL-10^+^ T cells among infiltrating T cells. Meanwhile, IL-17 and GM-CSF, which promote neuroinflammation (23, 37–39), were suppressed.

Given that IL-27 signaling in T cell leads to development of Tr1 cells through STAT1, AhR, and c-MAF transcription factors (13, 14, 16, 40), and that Tr1 cells secrete IL-10 to suppress autoimmune responses (40, 41), one would expect that peripheral tolerance requires IL-27 signaling in T cells. However, unexpectedly, we found that lack of IL-27 signaling in T cells did not affect induction of peripheral tolerance, as RAG1^−/−^ mice reconstituted either with WT or *Wsx*^−^*^/^*^−^ T cells were similarly susceptible to i.v. tolerance induction. Analysis of the T cells showed comparable frequencies of Tregs and similar production of inflammatory cytokines. This led us to conclude that IL-27 signaling in T cells is disposable for tolerance induction during ongoing inflammation. We speculate that IL-27 signaling in T is irrelevant for i.v. tolerance induction because effector T cells are resistant to the effects of IL-27 (42, 43). In our system, at the time when i.v. tolerance is induced, which is after EAE onset, encephalitogenic T cell responses have already developed and IL-27 would have no effect over these cells. The encephalitogenicity of *Wsx*^−^*^/^*^−^ CD4^+^ T cells has been previously addressed by our group and others (21, 44). It is known that these cells are more pathogenic than WT T cells. However, in this study we found that WT and *Wsx*^−^*^/^*^−^ mice developed a similar EAE course. This discrepancy may be due to differences in immunization protocols as in this study we used 200 µg of MOG_35–55_ and 240 ng of Pertussis toxin while others used 100 µg of peptide and 200 ng of toxin. We speculate that higher doses of MOG_35–55_ peptide in immunization inoculum could promote cell activation-induced death of *Wsx*^−^*^/^*^−^ CD4^+^ T cells or higher stimulation of Treg cells. Given that IL-27 induces tolerogenic DCs that suppress EAE (21), we next tested whether IL-27 signaling in DCs plays a role in i.v. tolerance induction.

It has been shown that DCs are pivotal for tolerance induction *via* i.v. and mucosal routes (3, 6, 31, 45). We found that in the CNS of EAE mice DCs express high levels of IL-27Rα, and that its expression is increased in mice subjected to tolerance induction. This observation suggests that DCs may be highly susceptible to IL-27 signaling or that they can respond to low concentrations of IL-27. The presence of DCs that express IL-27Rα rendered *Wsx*^−^*^/^*^−^ mice susceptible to tolerance induction, showing that IL-27 signaling in DCs, is necessary for establishment of peripheral tolerance to self-Ags during an autoimmune response. Further mechanistic analysis showed that disease amelioration correlated with a decreased frequency of IL-17- and GM-CSF-producing cells and a small increase in IL-10-producing T cells in the CNS. However, T cells were clearly skewed toward a Th1 lineage, as their IFN-γ production increased approximately threefold compared to PBS-treated mice. It has been shown that IFN-γ inhibits IL-17 production and exerts a protective role in EAE, and initial reports characterized IL-27 as a Th1-inducing cytokine (46, 47). These observations suggest that DC-induced T cell-derived IFN-γ may play a role in IL-27-dependent peripheral tolerance.

Our results show that IL-27 signaling in DCs contributes to peripheral tolerance induction in ongoing EAE and that the mechanisms of DC-mediated control of EAE include (i) suppression of IL-17 and GM-CSF and (ii) stimulation of Treg cells and IFN-γ/IL-10 production by T cells. STATs are a major family of kinases that translate cytokine signals to the nucleus of the cell and promote transcription of specific genes. STAT1 and -3 mediate IL-27 signal in T cells, leading to T-bet, c-MAF, and AhR expression and IL-10 production (13, 20, 40, 46). In the absence of STAT1, IL-27 has an inflammatory effect and supports differentiation of naive T cells into Th17 lineage (13). STAT1^−/−^ BMDCs remained tolerogenic after IL-27 treatment, as EAE severity was reduced in mice recipient of IL-27-treated BMDCs. Mascanfroni and colleagues showed previously that STAT3 is important for the effects of IL-27 signaling in DCs, as STAT3 is necessary for IL-27-induced expression of CD39, an ectonucleotidase (21). Apart from CD39, it has been shown that STAT3 also promotes IL-10 production in macrophages and Th17 cells (48, 49) and we observed that DCs from i.v. tolerized mice were proficient producers of IL-10, not IL-27, in the CNS. This led us to hypothesize that IL-27-induced DC-derived IL-10 plays a role in tolerance induction. In agreement with our hypothesis, lack of IL-10 production by DCs precluded the tolerogenic effect of IL-27, even though IL-17 production was suppressed. In this system, lack of disease amelioration correlated with reduced numbers of Tregs in i.v. tolerized mice, which shows that the mechanism of Treg cell expansion is dependent on IL-27-induced DC-derived IL-10.

Distinct subsets of DCs have been identified in mouse and human organs, and it is believed that each subset plays a specific role in inflammation and tolerance (26, 28, 30, 31, 50). In this context, it has been shown that mesenteric lymph node resident classical DCs (Lin^−^CD11c^+^CD64^−^MHC-II^+^, cDCs) are important for oral tolerance induction and that absence of MHC class II expression in mLN cDCs leads to colitis (51, 52). Using CD11b and CD103, we identified three distinct populations of classical DCs in the spleen of mice. Previous reports showed that CD103^+^ DCs in the gut are potent inducers of oral tolerance and stimulate Treg differentiation through TGF-β and retinoic acid (45, 53). Our data show that, on the one hand, CD103^+^ DCs readily uptake i.v. delivered Ag and that CD11b^+^CD103^+^ DCs from i.v. tolerized EAE mice were more efficient in inducing IFN-γ and IL-10. On the other hand, CD103^−^ and CD11b^hi^CD103^+^ DCs were more efficient in suppressing IL-17 production by naive T cells. Lack of IL-27R in DCs was sufficient to overcome this effect. IL-27 production was greater in cultures with CD103^−^ DCs from i.v. tolerized mice and this effect was not abrogated in the absence of IL-27Rα in DCs. It has been shown that IL-27 facilitates naive T cell differentiation into Th1 cells even though IL-27 does not induce IFN-γ production directly (19). Therefore, it would not be surprising if IL-27 acts on both T cells and DCs in the establishment of peripheral tolerance. Our data suggest that IL-27 skews DCs toward a Th1-inducing profile, which leads to suppression of Th17-driven inflammation, such as the case of EAE. Our data indicate that CD103^−^ DCs may be an initial source of IL-27 following intravenous administration of antigen, although more studies are needed to properly address this possibility.

Overall, our results indicate that an intricate network among DCs subsets results in IL-27-dependent tolerance induction in ongoing inflammation. These findings may indicate the need for research on the use of IL-27 as an adjunct factor for increasing the safety and efficacy of Ag-induced tolerance in harmful immune responses in clinical trials.

## Ethics Statement

Experimental protocols were approved by the Institutional Animal Care and Use Committee of Thomas Jefferson University.

## Author Contributions

Performed experiments: RT, JM, EM, JR, DH, and SY. Analyzed data: RT, BC, G-XZ, AR. Conceived and designed experiments: RT, G-XZ, and AR. Wrote the article: RT, BC, G-XZ, and AR. Supervised the study: AR.

## Conflict of Interest Statement

The authors declare that the research was conducted in the absence of any commercial or financial relationships that could be construed as a potential conflict of interest.

## Supplementary Material

Figure S1 Oral tolerance induction is IL-27-dependent. WT and *Wsx*^−^*^/^*^−^ mice (*n* of 5 per group) were immunized with MOG_35–55_ to induce EAE. At days 14, 16, and 18 p.i., mice were fed with 200 µg of MOG_35–55_ by gavage to induce tolerance. **(A)** Daily clinical scores and cumulative scores of disease severity. CNS-infiltrating cells numbers were determined by flow cytometry and hemocytometer on day 21 p.i. **(B)** Flow cytometry analysis of IL-10^+^, Foxp3^+^, IL-17^+^, GM-CSF^+^, and IFN-γ^+^ CD4^+^ T cells in the CNS from EAE mice described above. **(C)** Analysis of MHC-II, CD80, and CD86 expression by DCs. Bar graphs depict Mean ± SEM. Data are representative or pooled from three independent experiments with *n* of 5 mice per group with similar outcomes. **p* < 0.05 (Two-Way ANOVA with Bonferroni posttest).

Figure S2 STAT1-dependent and -independent mechanisms govern IL-27-induced tolerogenic DCs. Mature and immature WT and STAT1^−/−^ BMDCs were treated with IL-27 (20 ng/mL) for 18 h. **(A)** Expression of MHC-II, CD80 and CD86 was determined by flow cytometry. BMDCs were cultivated with WT naive CD4^+^ T cells and anti-CD3 (0.5 µg/mL) for 72 h at 37°C. **(B)** At the end of the culturing period, CD4^+^ T cells were analyzed by flow cytometry for intracellular cytokines. **(C)** Supernatants were assayed by ELISA. **(D)** WT and STAT1^−/−^ BMDCs (1.5 × 10^6^ cells/mouse) were i.v. transferred to WT recipient mice (*n* = 5/group) three days before immunization for EAE induction. **(D)** Daily clinical scores and cumulative scores of disease severity. CNS-infiltrating cells numbers were determined by flow cytometry and hemocytometer on day 21 p.i. **(E)** Flow cytometry analysis of IL-17^+^, GM-CSF^+^, IFN-γ^+^, IL-10^+^, and Foxp3^+^ CD4^+^ T cells in the CNS from EAE mice at day 21 p.i. Bar graphs depict Mean ± SEM. Data are representative from three independent experiments with similar outcomes. **p* < 0.05 [One-Way ANOVA in **(A,C)**; Two-Way ANOVA with Bonferroni posttest in **(D)**].
