# Supplementary figures and images for: Induction of Peripheral Tolerance in Ongoing Autoimmune Inflammation Requires Interleukin 27 Signaling in Dendritic Cells
Source: Front Immunol. 2017 Oct 27;8:1392. doi: 10.3389/fimmu.2017.01392 (PMC5663690; doi:10.3389/fimmu.2017.01392)

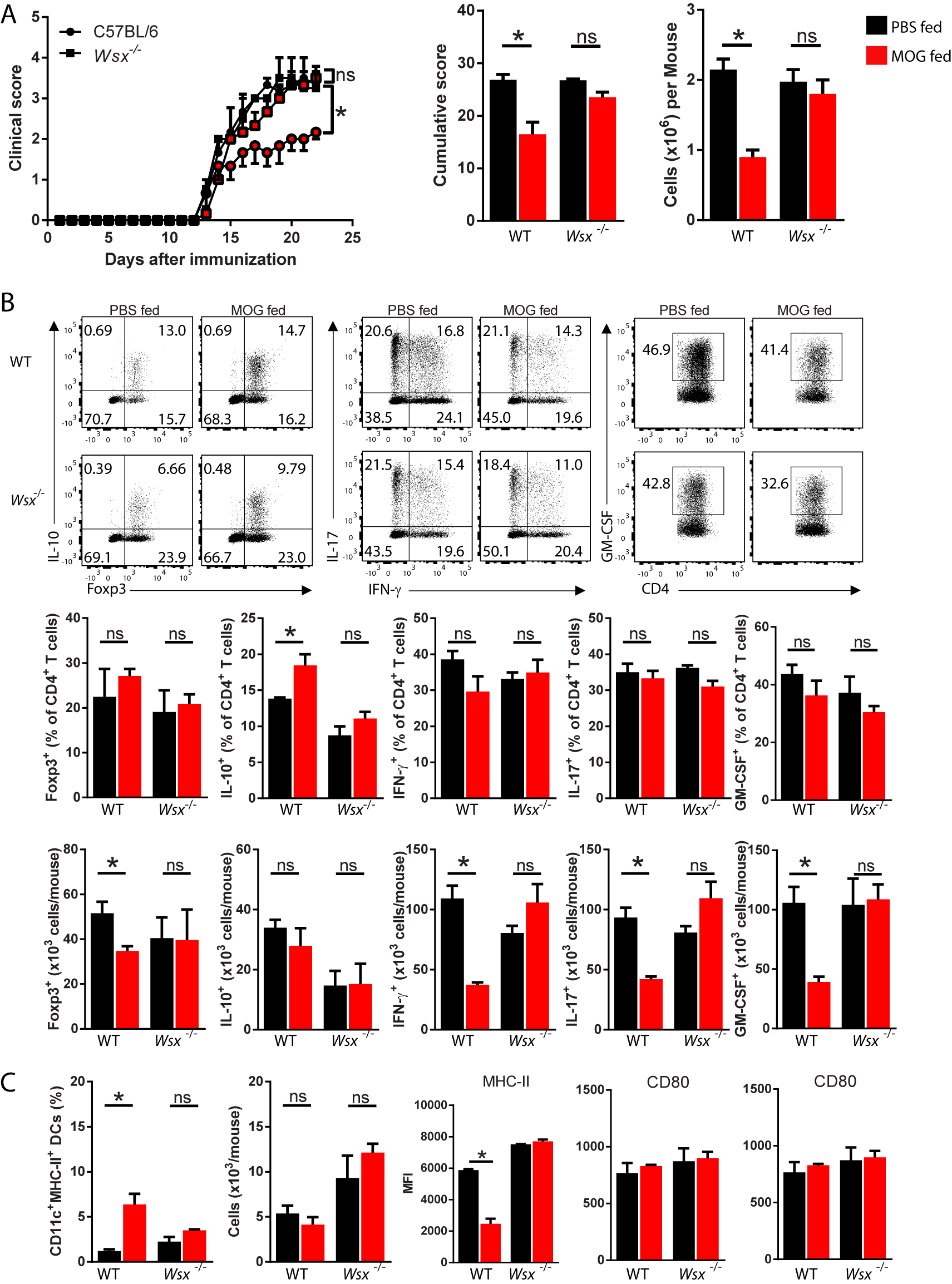

Supplement: Figure S1 — Oral tolerance induction is IL-27-dependent. WT and Wsx−/− mice (n of 5 per group) were immunized with MOG35–55 to induce EAE. At days 14, 16, and 18 p.i., mice were fed with 200 µg of MOG35–55 by gavage to induce tolerance. (A) Daily clinical scores and cumulative scores of disease severity. CNS-infiltrating cells numbers were determined by flow cytometry and hemocytometer on day 21 p.i. (B) Flow cytometry analysis of IL-10+, Foxp3+, IL-17+, GM-CSF+, and IFN-γ+ CD4+ T cells in the CNS from EAE mice described above. (C) Analysis of MHC-II, CD80, and CD86 expression by DCs. Bar graphs depict Mean ± SEM. Data are representative or pooled from three independent experiments with n of 5 mice per group with similar outcomes. *p < 0.05 (Two-Way ANOVA with Bonferroni posttest). [file image_1.tif]

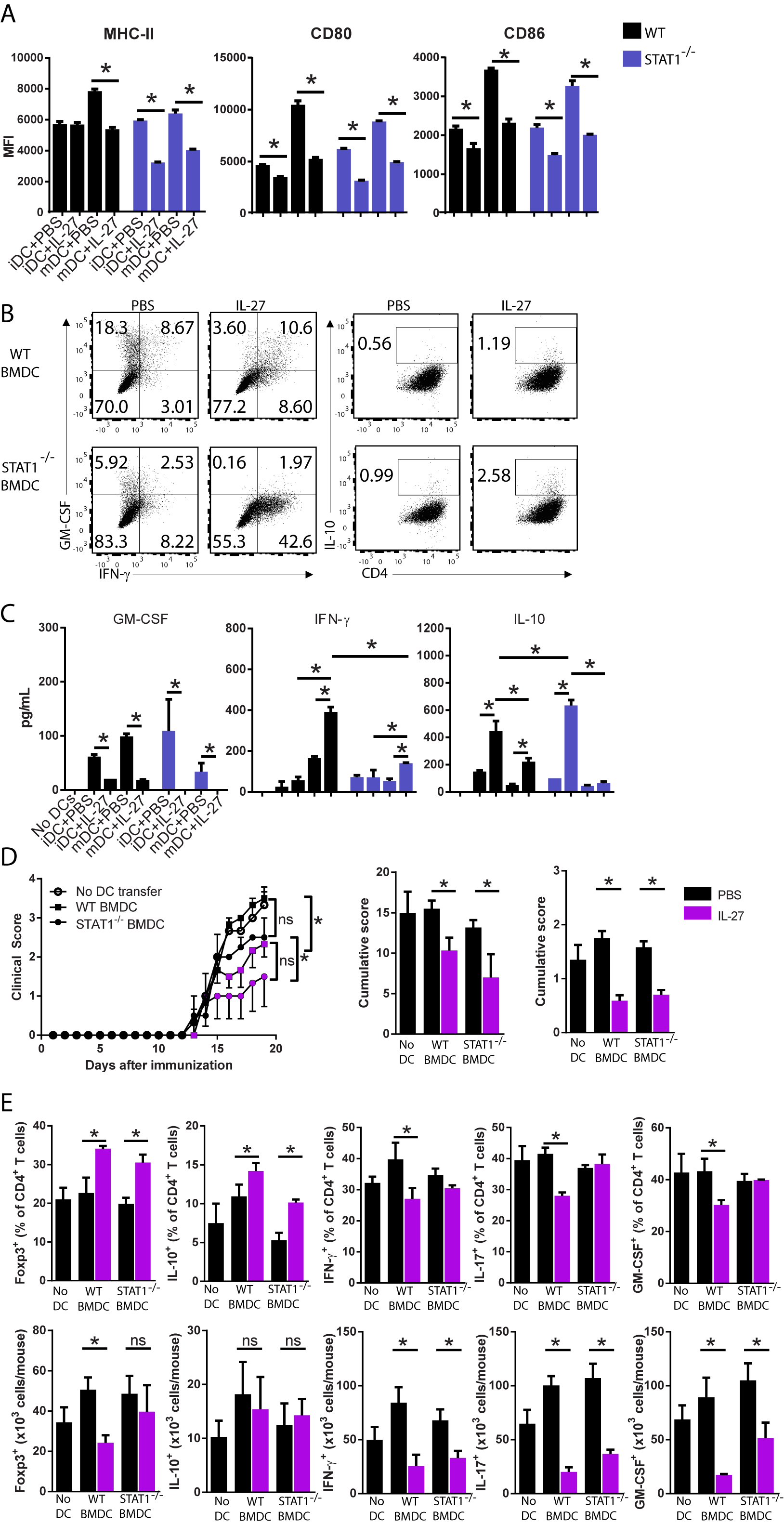

Supplement: Figure S2 — STAT1-dependent and -independent mechanisms govern IL-27-induced tolerogenic DCs. Mature and immature WT and STAT1−/− BMDCs were treated with IL-27 (20 ng/mL) for 18 h. (A) Expression of MHC-II, CD80 and CD86 was determined by flow cytometry. BMDCs were cultivated with WT naive CD4+ T cells and anti-CD3 (0.5 µg/mL) for 72 h at 37°C. (B) At the end of the culturing period, CD4+ T cells were analyzed by flow cytometry for intracellular cytokines. (C) Supernatants were assayed by ELISA. (D) WT and STAT1−/− BMDCs (1.5 × 10^6^ cells/mouse) were i.v. transferred to WT recipient mice (n = 5/group) three days before immunization for EAE induction. (D) Daily clinical scores and cumulative scores of disease severity. CNS-infiltrating cells numbers were determined by flow cytometry and hemocytometer on day 21 p.i. (E) Flow cytometry analysis of IL-17+, GM-CSF+, IFN-γ+, IL-10+, and Foxp3+ CD4+ T cells in the CNS from EAE mice at day 21 p.i. Bar graphs depict Mean ± SEM. Data are representative from three independent experiments with similar outcomes. *p < 0.05 [One-Way ANOVA in (A,C); Two-Way ANOVA with Bonferroni posttest in (D)]. [file image_2.tif]
